# Melanocortin Derivatives Induced Vascularization and Neuroglial Proliferation in the Rat Brain under Conditions of Cerebral Ischemia

**DOI:** 10.3390/cimb46030133

**Published:** 2024-03-05

**Authors:** Vasily V. Stavchansky, Vadim V. Yuzhakov, Larisa E. Sevan’kaeva, Natalia K. Fomina, Anastasia E. Koretskaya, Alina E. Denisova, Ivan V. Mozgovoy, Leonid V. Gubsky, Ivan B. Filippenkov, Nikolay F. Myasoedov, Svetlana A. Limborska, Lyudmila V. Dergunova

**Affiliations:** 1 National Research Center “Kurchatov Institute”, Kurchatov Sq. 2, Moscow 123182, Russia; ivmstalker@gmail.com (I.V.M.); filippenkov-ib.img@yandex.ru (I.B.F.); myasoedov-nf.img@yandex.ru (N.F.M.); limbor.img@yandex.ru (S.A.L.); dergunova-lv.img@yandex.ru (L.V.D.); 2 A. Tsyb Medical Radiological Research Center—Branch of the National Medical Research Radiological Center of the Ministry of Health of the Russian Federation, Koroleva Str. 4B, Obninsk 249036, Russia; yuzhvad.mail@yandex.ru (V.V.Y.); larisa.sevankaeva@mail.ru (L.E.S.); nkfomina@rambler.ru (N.K.F.); nastya93-rabota@mail.ru (A.E.K.); 3 Department of Neurology, Neurosurgery and Medical Genetics, Pirogov Russian National Research Medical University, Ostrovitianov Str. 1, Moscow 117997, Russia; dalina543@gmail.com (A.E.D.); gubskii@mail.ru (L.V.G.)

**Keywords:** tMCAO, ischemic stroke, melanocortin-like peptides, morphometry, arteriogenesis, neuroprotection

## Abstract

Stroke remains the second leading cause of death worldwide. The development of new therapeutic agents focused on restoring vascular function and neuroprotection of viable tissues is required. In this study the neuroprotective activity of melanocortin-like ACTH(4–7)PGP and ACTH(6–9)PGP peptides was investigated in rat brain at 24 h after transient middle cerebral artery occlusion (tMCAO). The severity of ischemic damage, changes in the proliferative activity of neuroglial cells and vascularization of rat brain tissue were analyzed. The administration of peptides resulted in a significant increase in the volume density of neurons in the perifocal zone of infarction compared to rats subjected to ischemia and receiving saline. Immunohistochemical analysis of the proliferative activity of neuroglia cells using PCNA antibodies showed a significant increase in the number of proliferating cells in the penumbra and in the intact cerebral cortex of rats receiving peptide treatment. The effect of peptides on vascularization was examined using CD31 antibodies under tMCAO conditions, revealing a significant increase in the volume density of vessels and their sizes in the penumbra after administration of ACTH(4–7)PGP and ACTH(6–9)PGP. These findings confirm the neuroprotective effect of peptides due to the activation of neuroglia proliferation and the enhancement of collateral blood flow.

## 1. Introduction

Ischemic stroke is a severe condition caused by the blockage of blood vessels in the brain by a thrombus or embolus, resulting in high rates of mortality and disability worldwide. As a result, there is a significant decrease in cerebral blood flow, which is accompanied by the emergence of neurological symptoms. Cerebral blood flow refers to the volume of blood that circulates through a specific amount of brain tissue within a given timeframe. Biomedical research in the field of stroke therapy focuses on timely detection of circulatory system disorders and the development of therapeutic interventions to minimize the consequences of vessel blockage. Currently, the primary treatment of ischemic stroke are thrombolytic treatment or mechanical thrombectomy [1]. While reperfusion is crucial to prevent neural death by restoring glucose and oxygen supply, the sudden restoration of blood flow after a stroke leads to oxidative damage to microvessels and worse ischemic damage [2]. Reperfusion leads to negative effects such as impairment of the blood brain barrier (BBB), extravasation of immune cells into the parenchyma of the nervous tissue, activation of the neuroglial pro-inflammatory phenotype [3,4]. Consequently, neurons, as well as vascular and glial cells of the penumbra surrounding the ischemic core, are at risk. Therefore, reperfusion treatment in stroke should be complemented with strategies regulating the inflammatory process, protecting penumbra neurons from death, and restoring vascular function and the integrity of the BBB. It is important to note that targeting individual components of the pathological process of ischemia-reperfusion (IR) is ineffective [5,6]. Therefore, comprehensive and time-coordinated therapeutic approaches are needed, including the use of molecules with pleiotropic properties that can affect various parts of postischemic recovery [7,8,9].

Potential neuroprotectors should be aimed at protecting nerve cells from damage and death, restoring vascular function, affecting glial, vascular and inflammatory cells, restoring nervous tissue and brain function after ischemia [10,11,12]. Promising candidates include peptide-based drugs possessing antioxidant, anti-inflammatory, regenerative, antiplatelet, and anti-apoptotic properties. These agents should demonstrate high efficacy, clear specificity, low immunogenicity, biocompatibility, gentle action, and absence of adverse effects [13,14,15]. In recent years, there has been active development of melanocortin-based drugs [16,17]. Melanocortins are a large family of neuropeptides that originate from a common precursor, the molecule called proopiomelanocortin. This molecule includes a group of melanocyte-stimulating hormones (α-, β-, γ-MSH) and adrenocorticotropic hormone (ACTH) [18,19]. Peptides based on ACTH and α-MSH exhibit neuroprotective effects against ischemic stroke [20,21]. Their mechanism of action involves antagonizing excitotoxic, inflammatory, and apoptotic pathways, which are primary mechanisms of damage during ischemia. Recent research indicates that animals treated with α-MSH following experimental hyperglycemia-induced stroke demonstrated increased survival rates and improved neurological outcomes. These effects were accompanied by a significant reduction in neuronal death, glial cell activation, oxidative stress, and nitrosative stress [20]. However, hormonal activity, a short half-life and the difficulty of delivering natural melanocortins to brain tissue contributed to the development of novel neuroprotectors, namely melanocortin derivatives. The TAT-HSA-α-MSH peptide, obtained by genetic fusion of α-MSH with the N-terminal 11 amino acid protein transduction domain of the human immunodeficiency virus (TAT) protein and human serum albumin (HSA), exhibits favorable pharmacokinetic properties and demonstrates effective penetration of BBB [22]. In addition, synthetic peptide Semax (Met-Glu-His-Phe-Pro-Gly-Pro) with a wide spectrum of neuroprotective activity was developed. Semax contains an ACTH(4–7) fragment, and the C-terminus is stabilized by the addition of the tripeptide Pro–Gly–Pro (PGP). Previous studies have demonstrated that Semax reduces the severity of neurological deficits and improves survival in animals following induced ischemic stroke [23]. Semax has shown neuroprotective properties and contributes to mitochondrial stability under stress induced by calcium ion flow deregulation [24]. Additionally, it has been found to inhibit nitric oxide synthesis [25]. The peptide also exhibits the ability to protect neuronal cell cultures from damage caused by oxidative stress and glutamate neurotoxicity [26,27], and has been shown to have an anticoagulant effect [28]. The effectiveness of Semax as treatment for patients at different stages of stroke has been demonstrated [29,30]. Specifically, the administration of Semax increased brain-derived neurotrophic factor (BDNF) plasma levels, accelerated functional recovery, and enhanced motor performance in post-stroke patients [30]. Previously, we carried out a series of transcriptomic studies using transient middle cerebral artery occlusion (tMCAO) model in rats [7,31,32,33,34,35]. We have shown that Semax primarily suppresses the expression of mRNAs of many genes involved in inflammation and activates a number of genes involved in the neurotransmitter system functioning in brain subcortical structures at 24 h after tMCAO [7]. Additionally, using immunodetection under tMCAO conditions, we have found that Semax has the ability to compensate for ischemia-impaired brain expression profiles of several key proteins involved in signaling pathways leading to inflammation and cell death [21].

Recently, a neuroprotective effect of another synthetic melanocortin-like peptide, ACTH(6–9)PGP (His–Phe–Arg–Trp-Pro-Gly-Pro), was discovered using cell culture studies [27,36]. Previous studies have shown that ACTH(6–9)PGP and Semax exhibit nootropic and anxiolytic activity [37].

The objective of this study was to conduct a morphofunctional investigation into the effects of Semax and ACTH(6–9)PGP peptides on the severity of ischemic damage, as well as on the neuroglial cell proliferation and tissue vascularization in the rat brain. As a result, consistent effect of both peptides was confirmed at the histological level in rats subjected to focal cerebral IR. We observed an activation of neuroglia proliferation, intensification of collateral blood flow and arteriogenesis, followed by the restoration of neuron morphology in peri-infarction zones of frontal cortex under tMCAO conditions. It is hypothesized that these events are involved in the mechanism of action of the investigated peptide drugs.

## 2. Materials and Methods

### 2.1. Animals and Experimental Groups

**Figure 1:**
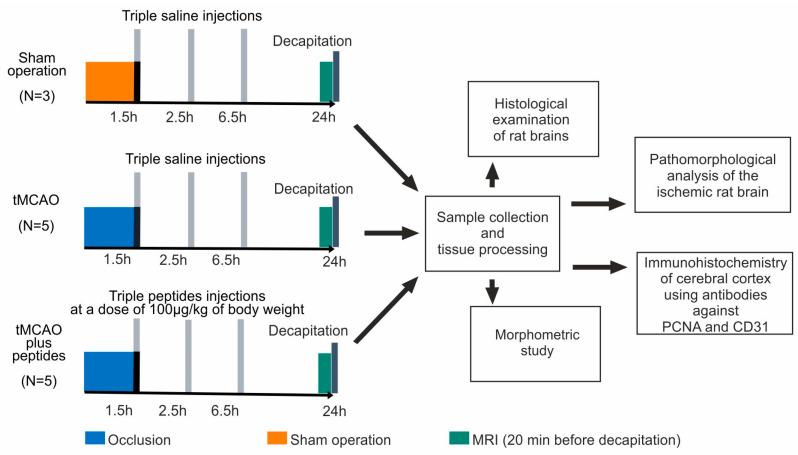
A scheme with the experimental design of our study.

Rats were obtained from the animal breeding house (AlCondi, Ltd., Moscow, Russia). White 2-month-old male Wistar rats (weighing 200–250 g) were maintained under natural condition with free access to water and food. The animals were divided into four groups: “sham operation” (SO), “ischemia–reperfusion” (IR), “ischemia–reperfusion and Semax administration” (IS), and “ischemia–reperfusion and ACTH(6–9)PGP administration” (IA). The IR, IS and IA experimental groups included 5 animals, whereas the SO group included 3 animals. A scheme with the experimental design is presented in Figure 1.

### 2.2. Rat Transient Middle Cerebral Artery Occlusion (tMCAO) Model

#### 2.2.1. Operation

The transient cerebral ischemia rat model was induced by endovascular occlusion of the right middle cerebral artery using a monofilament (Doccol Corporation, Sharon, MA, USA) for 90 min, followed by reperfusion for 22.5 h using the method of Koizumi et al. [38] with modifications [31]. The rats were decapitated 24 h after tMCAO. Prior to the surgical procedure and at the time of restoring blood flow, rats were anesthetized using 3% isoflurane with the EZ–7000 small animal anesthesia system (E-Z Anesthesia, Morrisville, NC, USA). Microsurgical clips were applied to the common carotid and internal carotid arteries, and two ligatures were applied to the external carotid artery. Then, an incision was made between the ligatures. The filament was inserted into the external carotid artery, guided into the internal carotid artery, and advanced to the origin point of the middle cerebral artery. A heating mat was used to maintain the animals’ body temperature during and after the surgery. The SO rats were subjected to a similar surgical procedure under anesthesia (neck incision and separation of the bifurcation), but without endovascular occlusion.

#### 2.2.2. Peptide Administration

The peptide (Semax or ACTH(6–9)PGP) was dissolved in saline at a concentration of 100 μg/mL and was injected at a dose of 100 μg/kg of body weight in rats of the IS and IA groups, respectively. Rats of the IR and SO groups were injected with saline. The volume of the injected solution depended on the weight of the animal, following a ratio of 1 μL per 1 g. The injections were intraperitoneal and were performed at 1.5, 2.5, and 6.5 h after tMCAO.

#### 2.2.3. Magnetic Resonance Imaging

The magnetic resonance imaging (MRI) study of the ischemic injury in rat brains was carried out using small animal 7T ClinScan tomograph (Bruker BioSpin, Billerica, MA, USA) as previously described [35]. For rats from the IR, IS and IA groups MRI was performed immediately before decapitation. MRI confirmed that all rats in the IR, IS and IA groups had a focal hemispheric lesion in the subcortex region of the right (ipsilateral) brain hemisphere and spread to the adjacent cortex.

### 2.3. Sample Collection and Tissue Processing

Rat brains were immersed in acidic Bouin’s solution for 24 h. A histotopographic cutting of the compacted brain was performed with a frontal orientation of 3 tissue blocks for subsequent cutting of the brain in the caudorastrial direction at the level of the frontal lobe, hippocampus—rostral lateral ventricle (RLV) and parietal lobe. Tissue samples of rat brains from each group were further fixed in acidic Bouin’s solution (total fixation time was 48 h) and washed with 70% ethanol.

Standard tissue processing was carried out using a Leica TP1020 Automatic Benchtop Tissue Processor (Leica, Wetzlar, Germany) (Supplementary Table S1). Oriented brain fragments were embedded in Paraplast medium (Leica, Wetzlar, Germany) at a HistoStar Embedding Workstation (Thermo Fisher Scientific, Waltham, MA, USA).

### 2.4. Histological Examination of Rat Brains

For morphological studies, serial sections of 5 µm thickness were obtained from paraffin blocks using a Leica RM2235 microtome (Germany). The sections were taken every 0.5 mm within the range of ischemic damage zones, from −1.5 to +1.5 from bregma. After dewaxing and hydration, the sections underwent hematoxylin and eosin (H&E) and Nissl staining.

For H&E staining, the sections were stained with hematoxylin for 5 min, followed by a 5 min wash with running water. Eosin staining was then performed for 0.5 min, followed by rinsing in distilled water. The sections underwent gradient alcohol dehydration, xylene transparency, and were sealed with balsam for microscopy. The H&E staining protocol was based on instructions provided by BioVitrum (St. Petersburg, Russia).

For Nissl staining, brain sections were immersed in a solution of 0.2% cresyl violet and 0.1% thionine (Fluka, Darmstadt, Germany) in a ratio of 3:1 for 30 min at room temperature. The sections were then washed in 96% alcohol and differentiated with hydrochloric acid alcohol for 5–10 s. After gradient alcohol dehydration, the sections were enclosed in balsam.

Histological sections were examined using a Leica DM 1000 microscope and photographed with a Leica ICC50 HD digital camera (Leica, Wetzlar, Germany) at four magnification levels: ×2.5, ×10, ×20, ×40. Scanned images of the preparations were obtained using a Nikon Super Coolscan 8000 ED digital scanner (Nikon, Tokyo, Japan) with a Nikon fh-g1 medical slide holder adapter. The optical resolution was set to at least 4000 dpi.

### 2.5. Pathomorphological Analysis of the Ischemic Rat Brain

A pathomorphological analysis was conducted, considering the morphological characteristics of normal and pathological variations of nerve cells. Additionally, artificial, supravital, and reactive changes in nerve tissue cells resulting from manipulations during brain isolation and immersion in fixative mixtures were considered [39,40,41]. Lesion areas were mapped histotopographically, and the precise determination of coronal slice levels was performed using a stereotactic atlas of the rat brain [42].

### 2.6. Immunohistochemistry

Immunohistochemical studies (IHC) of the cerebral cortex were conducted using polyclonal rabbit antibodies against the nuclear antigen of proliferating cells (PCNA) (PA5-27214, “Invitrogen”, Waltham, MA, USA, 1:100) and against a specific endothelial marker (CD31) (Anti-CD31 antibody [EPR17259], “Abcam”, Cambridge, UK, ab182981, 1:125). For rabbit antibody immunovisualization, Goat Anti-Rabbit IgG conjugated with horseradish peroxidase (Goat Anti-Rabbit IgG H&L (HRP), ab205718, “Abcam”, 1:1000) was used. Immunohistochemistry solutions were prepared in phosphate-buffered saline (PBS) buffer, pH 7.4 (ECO service, Moscow, Russia).

The detection of antigens on histological sections was performed following the standard requirements for immunoperoxidase methods. Before the experiments, all antibodies and kits were tested using the positive control of the experimental material. The optimal conditions for immunostaining were determined based on the ratio of the intensity of the immunopositive reaction to the “background”. According to the IHC protocol, deparaffinized sections were immersed in citrate buffer (pH 6.0), heated in a microwave oven (5 min, 720 W) and then hybridized with primary antibodies. Endogenous peroxidase was blocked in 3% hydrogen peroxide solution. The blocking buffer included 2% normal serum of animal donors of secondary antibodies, 1% bovine serum albumin and 0.1% Triton X-100. The sections were incubated overnight at 4 °C in a humid chamber with the primary antibody solutions.

After washing the samples in PBS (pH 7.4), secondary antibodies were applied to the sections for 1 h at room temperature. Substrate peroxidase was detected using diaminobenzidine (Liquid DAB+, DAKO, Santa Clara, CA, USA, K3468). Cell nuclei were counterstained with Mayer’s hematoxylin. The samples were washed with PBS, distilled water, then dehydrated in alcohols, cleared in xylene, and placed in Canadian balsam.

### 2.7. Morphometric Study

Quantitative studies were conducted following the basic principles of stereology in morphometry [43,44]. A computer analysis system was used for microscopic image analysis, utilizing a Leica DM 3000 microscope with a Leica DFC450 C digital camera (Leica, Wetzlar, Germany) and the Leica Application Suite, Version 4.9.0 licensed software package. Morphometric studies were conducted using three consecutive sections for each animal in the area of greatest ischemic damage. The sensorimotor area of the neocortex was divided into morphometric zones based on the atlas [42]. These morphometric zones are depicted in Supplementary Figure S1.

Scanned images of preparations stained with H&E were used to determine the area of the brain slice and the section area of the right hemisphere, including the size of the cortex and striatum. These were determined by contouring. Using serial sections stained with the Nissl method, the areas of undamaged tissue, perifocal zones in the cortex and striatum, and the sizes of infarct zones were calculated. This was achieved through image segmentation using an intensity threshold in a semi-automatic mode.

The following stereological and morphometric parameters were measured: St, the test area of 1 field (mm^2^); At, the total test area (mm^2^) (At = St × number of fields); Ai, the total cross-sectional area of i-structure (mm^2^); Vvi, the volume density of i-structures (%) (Vvi = Ai/At × 100%); Si, the average cross-sectional area of i-structure (µm^2^); Ns, the total number of cross-sections of i-structures per section area; Ni, the numerical density (Ni = Ns/At).

Morphometry of Nissl-stained neurons was conducted in layer V of the sensorimotor cortex (St = 0.3 mm^2^; 6 test fields at At = 1.8 mm^2^ for the undamaged cortex of each hemisphere, as well as 6 test fields at At = 1.8 mm^2^ for the penumbra). Only sections of the bodies of neurons that passed through their nuclei were considered. The quantitative characteristics of the neurons included the number of neurons per mm^2^, the average cross-sectional area of neurons (µm^2^) and volume density of neurons (%).

Quantitative analysis of the proliferative activity of neuroglia was conducted in layers II-VI of the cortex towards the corpus callosum (St = 0.3 mm^2^; 6 test fields at At = 1.8 mm^2^ for the intact cortex of each hemisphere; 6 test fields at At = 1.8 mm^2^ for the penumbra). The number of PCNA-positive nuclei was estimated.

Morphometry of vessels immunostained for CD31 was conducted in layers I-VI of the cortex up to the corpus callosum (St = 0.3 mm^2^; for each section, 10 test fields were analyzed at At = 3.0 mm^2^ for the intact cortex of the left and ipsilateral (right) hemispheres and 3 test fields for At = 0.9 mm^2^ for penumbra). Quantitative characteristics of the vessels included the volume density of vessels (%), average cross-sectional area of vessels (µm^2^) and the number of profile sections of vessels per mm^2^.

### 2.8. Data Analysis and Statistics

Statistical analysis of the results was performed using the Statistica 6.0 program (StatSoft, Inc., Tulsa, OK, USA). The nonparametric Mann–Whitney U-test was used to determine the significance of differences between groups for the measured variables. Differences were considered significant at *p* < 0.05. The data of quantitative characteristics of Nissl-stained neurons, proliferative activity of neuroglial cells during immunostaining for PCNA and vessels immunostained for CD31 was presented as median (Me) and error bars (lower quartile (LQ) and upper (UQ) quartile values). Dot plots were created using R (version 4.2.1 with libraries ggplot2 and cowplot). BioRender software (https://app.biorender.com/) was used for scientific imaging and illustration [45].

## 3. Results

### 3.1. MRI of Ischemic Foci after tMCAO

All rats had ischemic injury areas with ipsilateral hemispheric (subcortex plus cortex) localization in the brain of rats at 24 h after tMCAO. No pathological changes in the contralateral hemisphere of ischemic rats were observed according to the MRI data. Such injury localization was also typical for the rats after Semax and ACTH(6–9)PGP peptides administration at 24 h after tMCAO. T2 WI scan of rat brain, as well as quantitative assessment of the volume of ischemic brain injury at 24 h after tMCAO between IR, IS and IA groups using MRI are presented in Supplementary Figure S2.

### 3.2. Morphological Study of the Brain after Sham Operation (SO)

**Figure 2:**
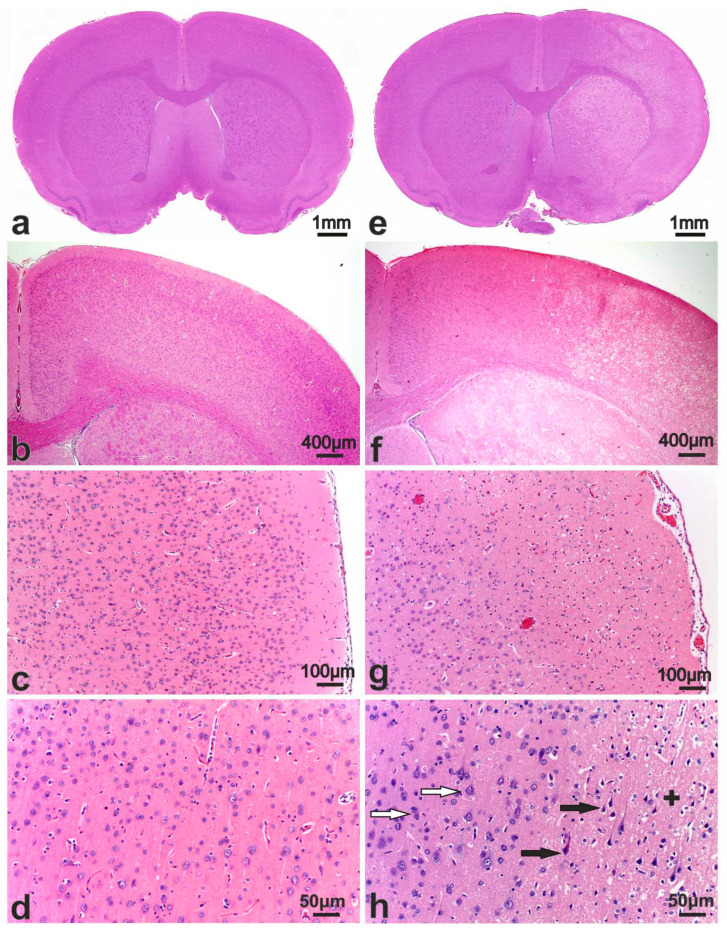
Photomicrographs of hematoxylin and eosin (H&E)-stained sections of the rat brains after sham operation (**a**–**d**) and at 24 h after tMCAO (**e**–**h**). Serial coronal brain sections at the levels of +0.7 (**a**) and +1.0 mm (**e**) from the bregma. (**b**,**f**) High magnification images of the dorsolateral regions of the right (ipsilateral) hemisphere at a level of +0.7 mm from bregma (**b**) and at a level of +0.48 mm from bregma (**f**). (**c**,**g**) Sensorimotor cortex of the right hemisphere. (**d**) Neurons in the pyramidal layer of the cortex of the right (ipsilateral) hemisphere. (**h**) Zone of transition from normal cortex through the penumbra to necrotic tissue at a level of +0.7 mm from bregma. White arrows show intact neurons. Black arrows show hypoxically injured neurons with pyknotic nuclei and pericellular edema. Neuropil edema is shown by plus (+).

**Figure 3:**
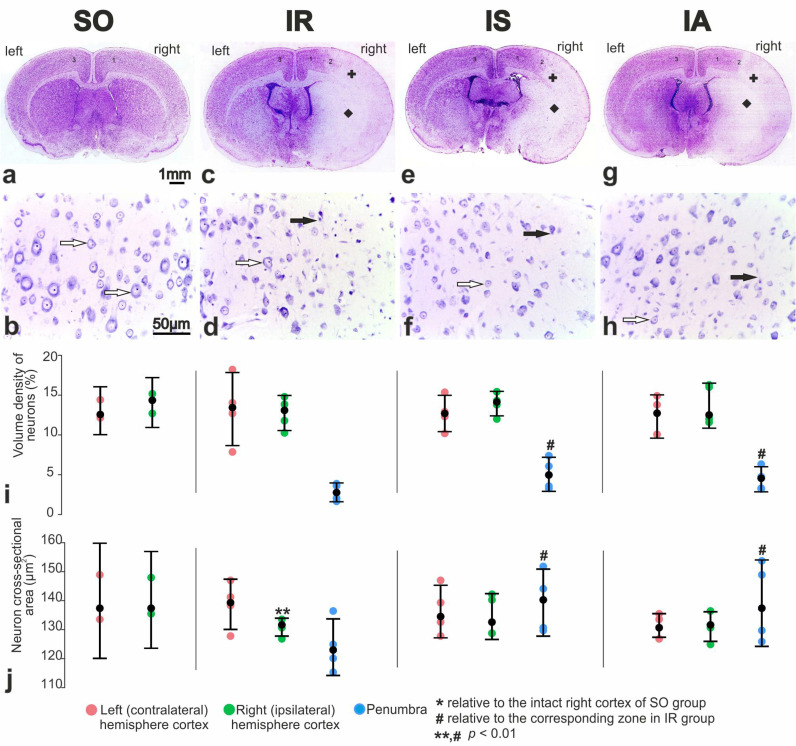
Photomicrographs of Nissl-stained sections of the rat brain after a sham operation (**a**,**b**), 24 h after tMCAO in the treatment with saline (**c**,**d**), Semax (**e**,**f**), ACTH(6–9)PGP (**g**,**h**). Frontal sections of the brain of a “sham operation” (SO) group (**a**), an “ischemia-reperfusion” (IR) group (**c**), an “ischemia-reperfusion + Semax” (IS) group (**e**) and an “ischemia-reperfusion + ACTH(6–9)PGP” (IA) group (**g**). Morphology of neurons in layer V of the sensorimotor cortex of ipsilateral (right) hemisphere in penumbra zone in rats of groups IR (**d**), IS (**f**), (IA) (**h**) and the corresponding zone in SO rats (**b**). Black arrows show chromatolysis in damaged neurons. White arrows show intact neurons. Infarction zones in cortex and striatum are shown by plus (+) and black diamond, respectively. The cortex of the right hemisphere, the penumbra, and the cortex of the left hemisphere are designated “1”, “2”, “3”, respectively. (**i**,**j**) Quantitative characteristics of Nissl-stained neurons in the pyramidal (V) layer. Volume density of neurons (%) (**i**), and neuron cross-sectional area (µm^2^) (**j**) were shown. Each of IR, IS, IA groups included five animals (n = 5). SO group included three animals (n = 3). Data are represented by dot plots using R statistics. The black dot denotes the median value of each group. The bottom and top of the vertical black bars represent the lower and upper quartile values, respectively.

In the microscopic examination of the brains of animals subjected to IR, SO rats were used as controls. On brain sections of SO group rats stained with hematoxylin and eosin (HE), the histological pattern of the cerebral cortex (Figure 2a–c), hippocampus, diencephalon, subcortical nuclei and neurogenesis zones in the rostral region of the lateral ventricles corresponded to normal variants. No pathological changes were observed in the neurons of the cortex (Figure 2d) and subcortical nuclei, thalamus, and nuclear formations of the diencephalon. In the hippocampus, neurons in the granular layer of the dentate gyrus and pyramidal layers in fields CA1–CA3 were arranged in an orderly manner. They had moderately basophilic cytoplasm, a rounded nucleus with granular chromatin, and a small nucleolus. In the neocortex, the neuropil had a fine-grained, fine-fibrous base, while in the diencephalon, it was weakly vacuolated. In frontal sections of the brain, the nerve fibers in the corpus callosum were densely packed, and numerous glial cells were visible between the fibers. In other parts of the brain, the white matter had a fibrous, fine-mesh structure, especially in the areas of the subcortical ganglia and pathways. Microscopic examination of frontal brain sections stained with Nissl staining revealed a histological pattern of the cerebral cortex and subcortical nuclei without any distinct features (Figure 3a). The neocortex showed six visible layers, consisting of cells of different sizes with a predominance of pyramidal neurons. Layers II and III of the cortex had similar morphological features and merged with each other in some areas. Layer IV was composed of small neurons located loosely or in groups. Layer V consisted of large pyramidal cells (Figure 3b). In preparations of the frontal lobe of the cerebral cortex, normochromic cells with a uniformly distributed chromatophilic substance in the cytoplasm were predominant in all layers. Their nuclei were round in shape, with large nucleoli usually located in the center of the nucleus. It is important to note that in brain sections of the SO group rats, certain vessels of the microvasculature appeared artificially “collapsed” due to the effects of the fixative, resulting in the formation of optically transparent perivascular spaces.

### 3.3. Pathomorphology of Focal Cerebral Ischemia and Morphofunctional Analysis of the Action of Peptide Drugs after tMCAO

Focal ischemic damage was observed as edema and areas of hypointensity in the caudal regions of the parietal lobe cortex in the ipsilateral hemisphere (Figure 2e,f). Microscopic examination revealed a collapse of the lumen of the capillaries and their desolation, rarefaction and brightening of neuropil due to edema and vacuolization, as well as the presence of numerous hyperchromic neurons with pericellular edema. In the level of hippocampus, the areas of developing stroke were expanded and visualized in caudoputamen, external capsule, and sensorimotor cortex. On the scanned sections, the maximum ischemic damage was observed in the area of the rostral lateral ventricle (RLV), ranging from −1.8 to +1.5 mm from bregma. The infarction zones covered almost the entire striatum and basolateral cortex extending to the fields of the somatosensory and motor cortex (Figure 2e–g). In all rats subjected to tMCAO conditions, the perifocal region was identified in the dorsolateral part of the frontal cortex and striatum. We also observed variations in the histotopographic location of ischemic zones, which appear to be attributed to the unique blood supply characteristics in rat brains. Intact areas were only identified in the dorsal region of the cortex (Figure 2f,h).

### 3.4. Morphofunctional and Morphometric Analysis of the Action of Peptide Drugs after tMCAO

The quantitative analysis revealed that in the IR group rats, swelling of the nervous tissue led to an increase by 11.2% in the ratio of the right hemisphere area to the left hemisphere area, compared to the SO group (Table 1). Additionally, the striatum area grew by 1.4 times (*p* < 0.05) (Table 1). However, rats treated with ACTH(6–9)PGP showed a statistically significant reduction in the relative infarct area to 54.2 ± 2.3% (*p* < 0.05) (Table 1), as well as a 2.8 times increase in the relative penumbra area in the cortex (*p* < 0.05) (Table 1), along with a pronounced tendency towards doubling the penumbra area in the striatum (*p* > 0.05) (Table 1). For animals treated with Semax, there was a trend towards a decrease in the relative area of the infarct zone in the right hemisphere, and an increase in the relative area of the penumbra in the right hemisphere cortex, compared to the data for animals in the IR group. The perifocal region in the dorsolateral part of the frontal cortex was selected for further morphofunctional investigation into the impact of Semax and ACTH(6–9)PGP peptides on the severity of ischemic damage, as well as the proliferation of neuroglial cells and vascularization of brain tissue in rats.

The infarct core which included the cortex, external capsule, and basal ganglia, consisted of nervous tissue with vascular endothelium destruction, neuropil vesiculation, and dying “pyknomorphic” neurons. Serial Nissl-stained sections showed a significant absence of neuron bodies in these areas compared to the sham operated animals (Figure 3a,c).

The perifocal zones of ischemia contained both neurons without pronounced pathological changes and nerve cells with signs of ischemic injury compared to SO (Figure 3b,d). Partial and total chromatolysis were observed in the cytoplasm of these neurons.

A typical histostructure of the rat brain in the “ischemia-reperfusion + Semax” (IS) group and the “ischemia-reperfusion + ACTH(6–9)PGP” (IA) group on the frontal section one day after tMCAO is shown in Figure 3e and Figure 3g respectively. Compared to the animals in the IR group (Figure 3c), the focal stroke area in the striatum and cortex was visually reduced, with expansion of intact cortex in the dorsal region of ipsilateral hemisphere. In layer V of the sensorimotor cortex and in the penumbra (Figure 3f), there was an increase in the number of normochromic pyramidal neurons. The administration of ACTH(6–9)PGP did not result in significant changes compared to rats treated with Semax (Figure 3f,h).

According to morphometric data, the volume density of neurons in layer V of the sensorimotor cortex ranged from 13.3% to 14.2% in both the left and right hemispheres of SO animals (Figure 3i). The numerical density of neurons in this region ranged from 960 to 1012 per mm^2^, with an average cross-sectional area of 141 µm^2^ (Figure 3j). In the dorsal region of the ipsilateral (right) cortex, the volume density and numerical density of neurons did not significantly change in the IR versus SO group (Figure 3a). However, the average area of their sections decreased to 131 µm^2^ (*p* < 0.05) (Figure 3j). In the perifocal region of the infarction, the volume density of neurons in the pyramidal layer decreased by 3.2 times (Figure 3i). Additionally, the numerical density of these neurons decreased by 2.9 times, along with a decrease in the size of neurocytes to 124 μm^2^ (Figure 3j).

After Semax administration, the volume density and sizes of neurons in the perifocal zone of the infarction increased by 1.5 times (Figure 3i) and 1.13 times (Figure 3j), respectively, compared to the IR group. There were no significant differences in the ipsilateral hemisphere of the brain for the studied parameters between the IS and IA groups (Figure 3i,j).

### 3.5. Immunohistochemical and Morphometric Study of the Effect of the Peptide Drugs on Proliferative Activity of Brain Neuroglial Cells under tMCAO Conditions

**Figure 4:**
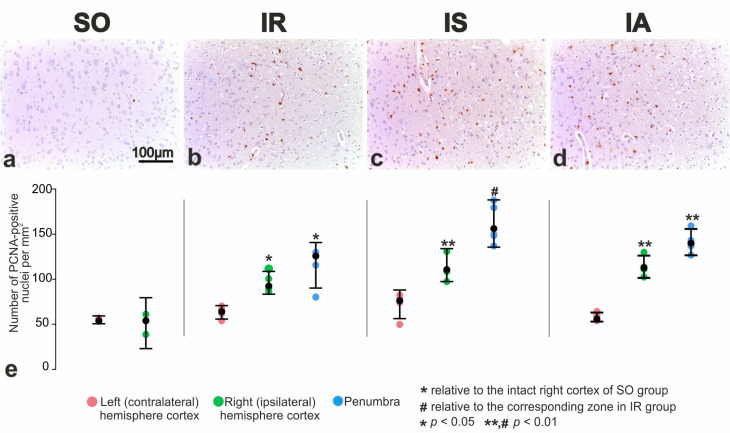
Proliferative activity of neuroglial cells in sensorimotor cortex of right hemisphere in rats of control SO (**a**), in penumbra of sensorimotor cortex in rats IR (**b**), IS (**c**) and IA (**d**) groups. Immunostaining for PCNA. Nuclei of proliferating cells were stained brown (**e**). Quantitative characteristics of the proliferative activity of neuroglial cells during immunostaining for PCNA. Number of PCNA-positive nuclei per mm^2^ were shown. Each of IR, IS, IA groups included five animals (n = 5). SO group included three animals (n = 3). Data are represented by dot plots using R statistics. The black dot denotes the median value of each group. The bottom and top of the vertical black bars represent the lower and upper quartile values, respectively.

To investigate the effect of Semax and ACTH(6–9)PGP peptides on the proliferative activity of brain cells after tMCAO, antibodies targeting proliferating cell nuclear antigen (PCNA) were utilized. Animals from the SO and IR groups were used as controls. In the region of the sensorimotor cortex (Figure 4a) and the striatum, a few neuroglial cells showed a positive reaction to PCNA.

The proliferation of neuroglia in the intact cortex of the ipsilateral hemisphere and in the perifocal region of infarction was significantly increased (Figure 4b). Histological examination of brain sections from the IS group stained for PCNA revealed the proliferative activity of neuroglia in the intact cortex of the ipsilateral hemisphere and in the perifocal region of infarction (Figure 4c). It was noteworthy that the use of Semax significantly activated the repopulation of both neuroglia and vascular endothelium in the intact tissues of the cortex and striatum in the ipsilateral hemisphere, particularly near the border with ischemic zones.

After PCNA immunostaining, brain samples from the IA group demonstrated IS-like effects. There was expansion of proliferation zones of neuroglial cells in the striatum, intact cortex of the ipsilateral hemisphere and in the penumbra (Figure 4d).

According to a morphometric study, in the sensorimotor cortex of the brain in SO rats a small number of cells showed a positive reaction to PCNA. In the dorsal zone, their numerical density was 51 to 55 per mm^2^ of the cortex area. In animals with ischemia, the numerical density of proliferating cells in the intact cortex significantly increased by 1.9 times (up to 96 ± 5 per mm^2^) compared to the SO rats (Figure 4e). Furthermore, the numerical density of proliferating cells in the penumbra reached 115 ± 9 per mm^2^ (Figure 4e). Under influence of Semax, the content of neuroglial cells with PCNA-positive nuclei significantly increased by 20% (*p* < 0.05) in the intact cortex of the ipsilateral hemisphere, and by 1.4 times in the perifocal area of the infarction (*p* < 0.01) in comparison of IS vs. IR (Figure 4e). Similar changes were observed after the administration of the ACTH(6–9)PGP peptide. The number of proliferating cells significantly increased by 16% and 20% in the intact cortex of the ipsilateral hemisphere and in the penumbra, respectively (Figure 4e).

### 3.6. Immunohistochemical and Morphometric Study of the Effect of the Peptides on Cerebral Cortex Vascularization after tMCAO

**Figure 5:**
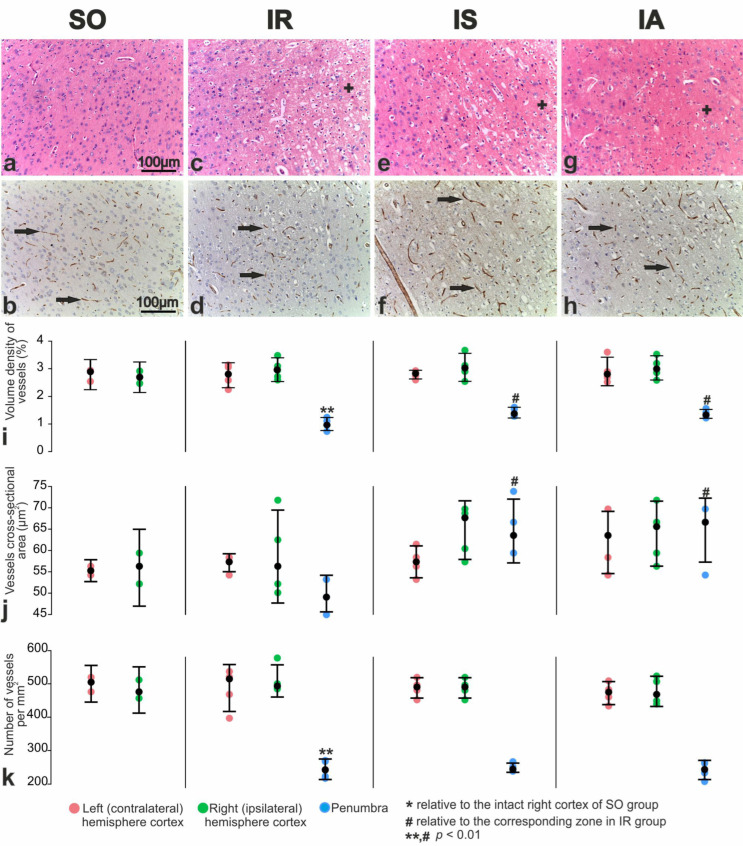
Morphology of vessels and neurons of the sensorimotor cortex of the right (ipsilateral) hemisphere of SO (**a**,**b**), IR (**c**,**d**), IS (**e**,**f**), and IA (**g**,**h**) rats. H&E staining (**a**,**c**,**e**,**g**); CD31 immunostaining with additional staining with hematoxylin (**b**,**d**,**f**,**h**). Black arrows show microvessels, neuropil edema is shown by a plus sign (+). (**i**–**k**) Quantitative characteristics of vessels immunostained for CD31. Volume density of vessels (%) (**i**), vessel cross-sectional area (µm^2^) (**j**), and the number of neurons per mm^2^ (**k**) were shown. Each of IR, IS, IA groups included five animals (n = 5). SO group included three animals (n = 3). Data are represented by dot plots using R statistics. The black dot denotes the median value of each group. The bottom and top of the vertical black bars represent the lower and upper quartile values, respectively.

Antibodies against the cluster of differentiation 31 (CD31) protein were used to investigate the impact of Semax and ACTH(6–9)PGP peptides on cerebral cortex vascularization 24 h after tMCAO. We observed that microcirculatory vessel profiles (arterioles, capillaries and venules) were distributed relatively evenly in H&E-stained sections and CD31-immunostained brain samples of SO rats (Figure 5a,b).

In the microvasculature of the cortex in the ipsilateral hemisphere of IR animals, there was a collapse and desolation of the capillary lumen, swelling of the vascular endothelium and perivascular edema. Individual vessels appeared dilated, overflowing with blood. The number of microvasculature vessels was significantly reduced (Figure 5c,d).

At the frontal section of rat brains from the IS group, Semax administration resulted in increased arterioles in intact areas of the cortex, particularly at the border with the penumbra. In the perifocal zone of infarction, there was an increase in the number of histologically normal neurons (Figure 5e) and intact microcirculatory vessels (Figure 5f).

H&E-stained and CD31-immunostained brain samples from the IA group showed no significant changes in the dorsal zones of the sensorimotor area of the contra- and ipsilateral hemispheres compared to the IS samples. There was an expansion of intact cortex in the dorsal region of the ipsilateral hemisphere, an increase in vascular abundance at the border with the perifocal zone of infarction and less pronounced hypoxic damage to penumbra neurons compared to IR (Figure 5g,h).

The volume density of vessels in the sensorimotor cortex of the left and right brain hemispheres SO rats was 2.79% and 2.70%, respectively (Figure 5i). Additionally, the average cross-sectional area of these vessels was 55 and 56 µm^2^, respectively (Figure 5j), and the numerical density of vessels in the same brain areas was 500 and 481 per mm^2^ (Figure 5k).

Under IR conditions, the volume density and numerical density of vessels in the intact areas of the cortex remained significantly unchanged compared to the SO control (Figure 5i,j). In the perifocal region of the infarction, the volume density of vessels significantly decreased by 2.4 times (Figure 5i), while the numerical density of vessels decreased by 2.2 times (Figure 5k). As a result, cross-sectional area of vessels reached 50 µm^2^ (Figure 5j). After Semax administration in animals of the IS group, the volume density and sizes of vessels were increased by 1.25 times (*p* < 0.01) (Figure 5i,j) and by 1.28 (Figure 5i,j), respectively in the perifocal zone of infarction in IS versus IR groups. Semax-like changes were observed after ACTH(6–9)PGP peptide administration (Figure 5i,j).

## 4. Discussion

For many years, animal models of cerebral ischemia have been used to understand the molecular mechanisms of drugs used in stroke treatment. The tMCAO model which ensures reproducibility and adherence to scientific standards, is considered the most representative of the clinical picture of transient ischemia, and damage occurring when blood flow is restored with thrombolytic agents [46,47]. Depending on the specific conditions of the tMCAO model, the resulting lesion can be either localized in the subcortical structures of the brain (subcortical localization) or extends to the cortex (hemispheric localization). In this study, we utilized the tMCAO model to induce a reproducible hemispheric (cortex plus subcortical structures) localization of ischemic damage involving the basolateral region of the cortex and basal nuclei of the ipsilateral hemisphere in the rat brain. Using MRI, we detected the location and volume of ischemic foci in animals after tMCAO. Histological analysis conducted 24 h after occlusion revealed classical zones of ischemic infarction with vascular disorders and severe destructive changes in nerve cells within the blood supply zone of the right middle cerebral artery. The results of comprehensive morphofunctional studies provided objective evidence to confirm the similar neuroprotective effect of the two melanocortine-related peptides: Semax and ACTH(6–9)PGP.

Classically, the penumbra is defined as the area surrounding the ischemic nucleus, which experiences metabolic and functional impairments but remains viable. Many years ago, it was discovered that in the ischemic penumbra, numerous neurons remain structurally intact but functionally inactive [48]. When reperfused within the therapeutic window, neurons in this region still retain the potential for complete recovery. Modern approaches to stroke treatment drug development are based on this concept. Numerous studies on the neuroprotective properties of potential drugs are conducted in the peri-infarct region adjacent to the infarct core [49,50,51]. In this study, in rats treated with ACTH(6–9)PGP, we observed a significant reduction in the relative infarct area and an expansion in the relative penumbra area within the cerebral cortex when compared to the IR group. Concurrently, Semax tended to exhibit a similar influence. As a result, we focused on studying the neuroprotective properties of the peptides in the peri-infarction region, which is localized in the frontal cortex of rats. In the perifocal region of the rat brain, both peptides significantly increased the number of normochromic neurons as well as the volume density of neurons compared to the saline treated IR control (Figure 3). The observed neuroprotective effect of Semax and ACTH(6–9)PGP peptides during tMCAO conditions may be attributed to their ability to protect neuronal cell cultures from damage caused by oxidative stress and glutamate neurotoxicity [24,26,27,36]. According to Novosadova et al., the number of human NPCs was approximately 15% higher under the action of Semax compared to the control [26]. These NPCs were derived from induced pluripotent stem cells (iPSCs) that survived after oxidative stress caused by hydrogen peroxide [26]. Moreover, Semax demonstrated an approximately 40% increase in the number of surviving cells in differentiated cultures enriched with dopaminergic neurons when exposed to peroxide, compared to cultures without the peptide. Additionally, in conditions of glutamate neurotoxicity, Semax enhanced the survival of cerebellar granule neurons by an average of 30%, as reported by other researchers [24]. The authors proposed that the neuroprotective effect of Semax is attributed to the peptide’s influence on calcium homeostasis and the functional status of mitochondria. Furthermore, recent studies have also demonstrated the extensive neuroprotective activity of ACTH(6–9)PGP [27,36]. The effects of the ACTH(6–9)PGP peptide on the survival of the cultured cortical neurons under the excitotoxic effects of glutamate were investigated [27]. It was observed that the peptide, depending on dosage, provided protection against cell death and significantly increased the number of neurons that restored calcium homeostasis after glutamate withdrawal [27]. The neuroprotective effect of ACTH(6–9)PGP was accompanied by a decrease in the development of delayed calcium dysregulation and synchronous depolarization of mitochondria. In another study, the ACTH(6–9)PGP peptide demonstrated dose-dependent protection of neuroblastoma cells from the SH-SY5Y line against oxidative stress induced by hydrogen peroxide, tert-butyl hydroperoxide, or potassium cyanide. Additionally, the peptide exhibited proliferative activity in SH-SY5Y cells, acting by modulating NF-κB target genes associated with proliferation and stimulating the prosurvival NRF2-gene-related pathway, while also reducing apoptosis [36].

Moreover, using PCNA-immunostaining, an increase in the proliferation of cells in intact areas of the cortex of the ipsilateral (right) hemisphere and in the perifocal region of the infarction was observed in IR versus SO pairwise comparison (Figure 4). In previous studies by Imai et al., the presence of PCNA-immunopositive cells in the peri-infarct brain zone 24 h after tMCAO has been observed [52]. Double-label immunofluorescence using microglial response factor-1 (MRF-1) revealed that the majority of PCNA immunoreactive cells in the peri-infarct zone were microglia. GFAP-positive astrocytes did not exhibit PCNA-positive staining during the 24-h post-ischemic period. Thus, it can be inferred that the PCNA-immunostained cells found in the penumbra also primarily consist of microglia. Activated microglia are recruited to the penumbra, where they play a crucial role in vascular repair and maintenance of BBB integrity [53,54,55]. It was shown that during the first 24 h after a stroke, activated microglia predominantly have an anti-inflammatory M2 phenotype [56,57,58,59]. In the later stages of damage, the phenotype of microglial cells becomes proinflammatory [60]. The protective effect of microglia during the acute phase of stroke (less than 24 h) has been indirectly demonstrated by the exacerbation of neuronal damage when microglial depletion occurs after ischemic stroke [61,62,63]. These cells also promote neurogenesis and angiogenesis by producing nerve and vascular growth factors [64,65]. The administration of Semax or ACTH(6–9)PGP peptides showed an additional activating effect on the proliferative process, significantly increasing the number of PCNA-immunostained cells. The activation of cell proliferation observed in our study indicates a positive effect of peptides on their microglial environment in the perifocal areas of stroke.

CD31 antibodies, widely used as an endothelial marker, are used to immunostain existing and developing brain blood vessels under both physiological and pathological conditions [66]. The results of our immunohistochemical studies using the endothelial marker CD31 showed a significant increase in the intact vessels number of the microcirculatory bed in the perifocal region of the infarction when the studied peptides were used (Figure 5). Additionally, an increase in vascular filling was observed in the neocortex of the ipsilateral hemisphere. However, there were no significant microscopic signs of neoangiogenesis in the penumbra after administration of both peptides compared to saline at 24 h after tMCAO. According to modern concepts, the vasculature plays a key role in the development of the pathological process caused by IR [2]. The vasculature remains disrupted in small arterioles and capillaries even after blood flow is restored in a large artery [67,68]. Several studies have shown that during the first day, the early phase of cerebral blood volume recovery is associated with improved collateral blood flow (arteriogenesis) [69,70,71]. Arteriogenesis occurs through pre-existing non-functioning arteriolar anastomoses and due to an increase in the diameter of existing arterial vessels [72,73]. The condition of collaterals and the adequacy of capillary perfusion appear to be the best predictors of clinical outcome after stroke and can be considered as potential therapeutic targets [9,74].

**Figure 6:**
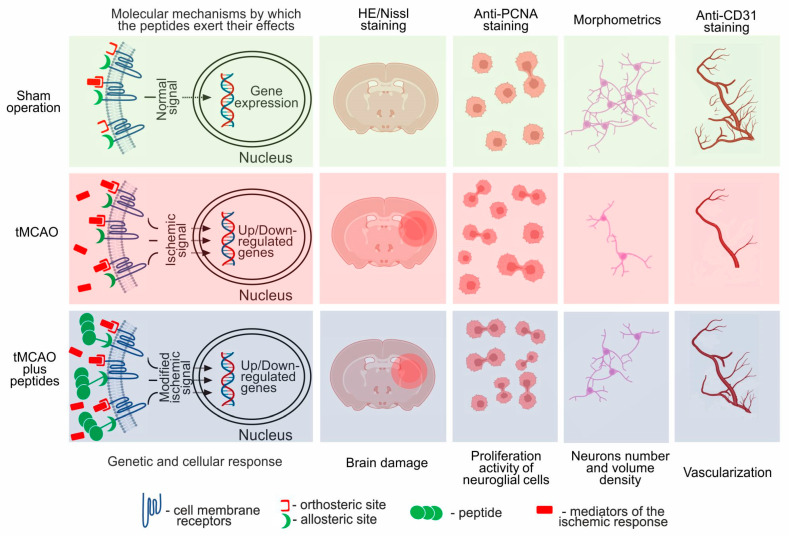
Model explaining the molecular and cellular mechanisms by which the Semax and ACTH(6–9)PGP peptides induced vascularization and neuroglial proliferation in the rat brain under conditions of cerebral ischemia. The illustration was made using BioRender.

It is known that the mechanisms of action of melanocortins are determined by their specific ligand-receptor interactions on the plasma membranes of target cells [75,76,77]. However, the involvement of melanocortin receptors in the binding of Semax and ACTH(6–9)PGP peptides has not been demonstrated to date. Experimental data on the specific binding of these peptides have suggested the allosteric effect of peptides on various receptor systems [78]. Given these data, we propose a model of peptide regulation in the brain (Figure 6). According to our hypothesis, the allosteric interaction of the peptide with various types of receptors, together with hormones and mediators of the ischemic response, can influence gene expression, modulating disorders caused by ischemia.

Previously, using high-throughput RNA sequencing (RNA-Seq), we identified a genome-wide mRNA expression profile in subcortical brain samples of SO, IR and IS rats [7]. As a result, we found hundreds of differentially expressed genes (DEGs) under IR and Semax treatment at 24 h after tMCAO. Among them, there were a number neurogenesis-, angiogenesis- and growth factor-related DEGs. Additionally, gene-encoded protein kinases (*Prkca*, *Ptk2b*), as well as neuronal factors (*Neurod6*, *Ngef*) and neurotrophic factors (*Ntrk1*, *Ntrk2*, *Ntrk2*) were upregulated under IR conditions after administration of Semax compared to saline (Supplementary Table S2). Furthermore, using RNA-Seq, we revealed 131 and 322 DEGs to the Semax and ACTH(6–9)PGP peptide at 4.5 h after tMCAO, respectively, in dorsolateral areas of the frontal cortex of rats [32]. Both peptides can partially prevent changes in the immune- and neurosignaling-related gene expression profiles disturbed by the action of ischemia at 4.5 h after tMCAO. Concurrently, ACTH(6–9)PGP upregulates DEGs of growth factors (*Bdnf*, *Fgf9*, *Egr1*, *Egr2*, *Egr3*, *Egr4*), as well as neurogenesis-associated genes (*Adcyap1*, *Ifrd1*, *Chn1*) at 4.5 h after tMCAO [32]. Thus, at the transcriptomic level, we found similarities and differences in the effects of Semax and ACTH(6–9)PGP in the early post-stroke period. However, our study was limited by the absence of genome-wide data related to Semax and ACTH(6–9)PGP action in the dorsolateral regions of the brain at 24 h after tMCAO. A limitation of our work is also the lack of studies using specific markers for different cell types, which prevents us from accurately classifying proliferating cells.

Thus, we firstly found that both Semax and ACTH(6–9)PGP peptides induced vascularization and neuroglial proliferation in the rat brain under conditions of cerebral ischemia. This result is the strength of our study. Therefore, we believe that the key mechanisms underlying neuroprotective action of both melanocortin-like peptides in acute transient cerebral circulation disorders in rats are the activation of neuroglia proliferation, protection of microcirculatory vessels from damage, intensification of collateral blood flow and arteriogenesis, as well as restoration of neuron morphology in penumbra. Whether there are any differences in the effects of Semax or ACTH(6–9)PGP between smaller or larger lesions remains an open question. It should be noted that our study is limited by the lack of extrapolation of results from the rat model to humans. However, we believe that the results obtained in the tMCAO model provide valuable insights into the mechanism of action of the studied peptides at the tissue and cellular levels. These findings should be considered in future clinical studies.

## 5. Conclusions

Morphological, immunohistochemical, and morphometric study revealed that two melanocortin-like peptides, Semax and ACTH(6–9)PGP, induce proliferative activity of neuroglial cells and vascularization of brain tissues in the perifocal zones. This study serves as a basis for identifying the mechanism of action of melanocortin-like peptides at the cellular level. By conducting further research on transcriptomic and proteomic levels of gene expression regulation in different models of ischemia, we can gain a better understanding of the mechanisms of peptide action at the molecular level. The results can expand frontiers of potential therapeutic applications of peptides based on identified molecular signatures of peptide-induced neuroprotection in stroke. Additionally, further studies on microglial phenotype switch under those therapy options would be an interesting future research goal.

## Figures and Tables

**Table 1:** Quantitative morphological indices of the rat brain across “sham operation” (SO) “ischemia–reperfusion” (IR), “ischemia–reperfusion and Semax administration” (IS), “ischemia–reperfusion and ACTH(6–9)PGP administration” (IA) groups.

Index	SO	IR	IS	IA
Area of the frontal brain section at the level of the rostral part of the lateral ventricles (mm^2^)	71.1 ± 0.8	76.5 ± 5.1	76.7 ± 0.7	82.6 ± 3.4
Area of the right hemisphere (mm^2^)	35.7 ± 0.5	40.1 ± 2.3	43.2 ± 0.7	45.0 ± 2.1
Percentage of the total area that the right hemisphere occupies (%)	50.3 ± 0.4	52.6 ± 0.9	55.5 ± 1.1	54.5 ± 1.4
Percentage comparison of the right hemisphere area to the left hemisphere area (%)	100.1 ± 0.4	111.2 ± 4.2	125.4 ± 5.9	120.6 ± 6.9
Area of the infarction zone in the right hemisphere (mm^2^)		25.5 ± 1.1	25.5 ± 1.5	24.5 ± 1.9
Percentage of the total area that the infarction zone in the right hemisphere occupies (%)		64.0 ± 2.6	59.0 ± 3.3	54.2 ± 2.3 #
Area of the right hemisphere cortex (mm^2^)	17.1 ± 0.1	19.9 ± 1.2	20.0 ± 0.8	20.1 ± 0.6
Percentage of the intact cortex area in the right hemisphere (%)		18.9 ± 1.4	20.1 ± 1.9	22.1 ± 3.0
Percentage of the infarction area in the right hemisphere cortex (%)		75.6 ± 2.8	71.7 ± 3.9	58.1 ± 8.2
Percentage of the penumbra area in the right hemisphere cortex (%)		5.4 ± 1.6	8.2 ± 2.4	15.2 ± 3.9 #
Area of the right hemisphere’s striatum (mm^2^)	8.9 ± 0.1	12.5 ± 0.9 *	13.3 ± 0.8	10.3 ± 0.6
Percentage of the intact striatum area in the right hemisphere (%)		1.3 ± 0.9	1.0 ± 0.6	1.5 ± 0.9
Percentage of the infarction area in the striatum of the right hemisphere (%)		89.1 ± 4.9	88.3 ± 4.5	79.1 ± 5.1
Percentage of the penumbra area in the striatum of the right hemisphere (%)		9.6 ± 4.6	10.7 ± 4.0	19.4 ± 5.0

The data are presented as the mean ± standard error of the mean. The significant differences related to the SO group (*p* < 0.05) are marked by an asterisk (*), whereas significant differences related to the IR group (*p* < 0.05) are marked by a hashtag (#).

## Data Availability

RNA-sequencing data have been deposited in the Sequence Read Archive database under accession code SRP148632 (SAMN09235828-SAMN09235839) [79], and PRJNA491404 (SAMN10077190-SAMN10077195) [80].

## Supplementary Materials

The following supporting information can be downloaded at: https://www.mdpi.com/article/10.3390/cimb46030133/s1, Figure S1: The morphometry zones of Nissl-stained neurons; Figure S2: MRI of ischemic foci after tMCAO; Table S1: The histological wiring of rat brain samples; Table S2: Upregulated DEGs associated with neurogenesis, angiogenesis, and growth factors activity in IS vs. IR pairwise comparison.
