# Supplementary material for: Melanocortin Derivatives Induced Vascularization and Neuroglial Proliferation in the Rat Brain under Conditions of Cerebral Ischemia
Source: Curr Issues Mol Biol. 2024 Mar 5;46(3):2071–92. doi: 10.3390/cimb46030133 (PMC10969580; doi:10.3390/cimb46030133)
Supplement: Supplementary file 1 [file cimb-46-00133-s001.zip › Supplementary Figure S1.pptx]

## Slide 1
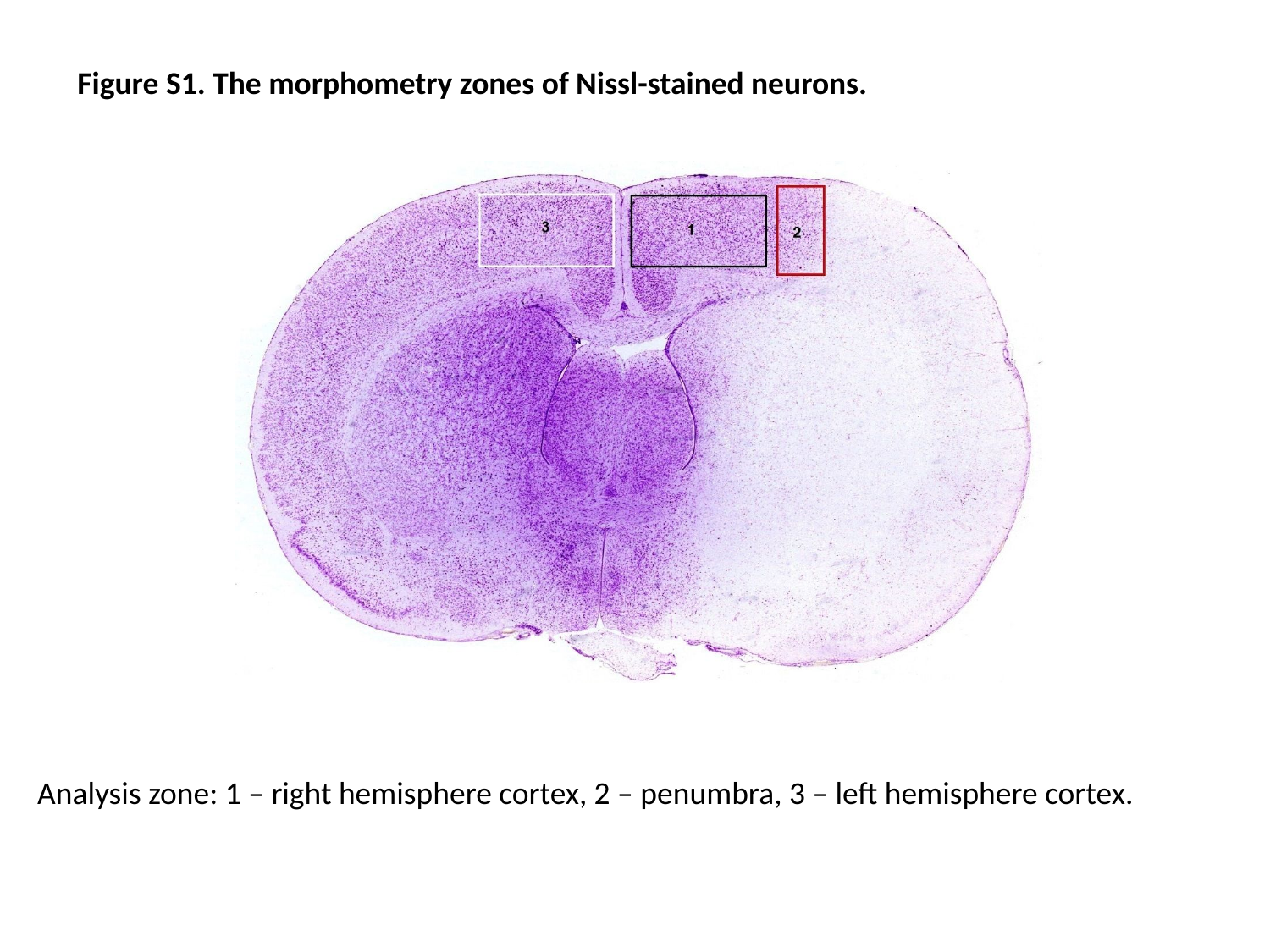

Figure S1. The morphometry zones of Nissl-stained neurons.
Analysis zone: 1 – right hemisphere cortex, 2 – penumbra, 3 – left hemisphere cortex.
