# Supplementary material for: Melanocortin Derivatives Induced Vascularization and Neuroglial Proliferation in the Rat Brain under Conditions of Cerebral Ischemia
Source: Curr Issues Mol Biol. 2024 Mar 5;46(3):2071–92. doi: 10.3390/cimb46030133 (PMC10969580; doi:10.3390/cimb46030133)
Supplement: Supplementary file 1 [file cimb-46-00133-s001.zip › Supplementary Figure S2.pptx]

## Slide 1
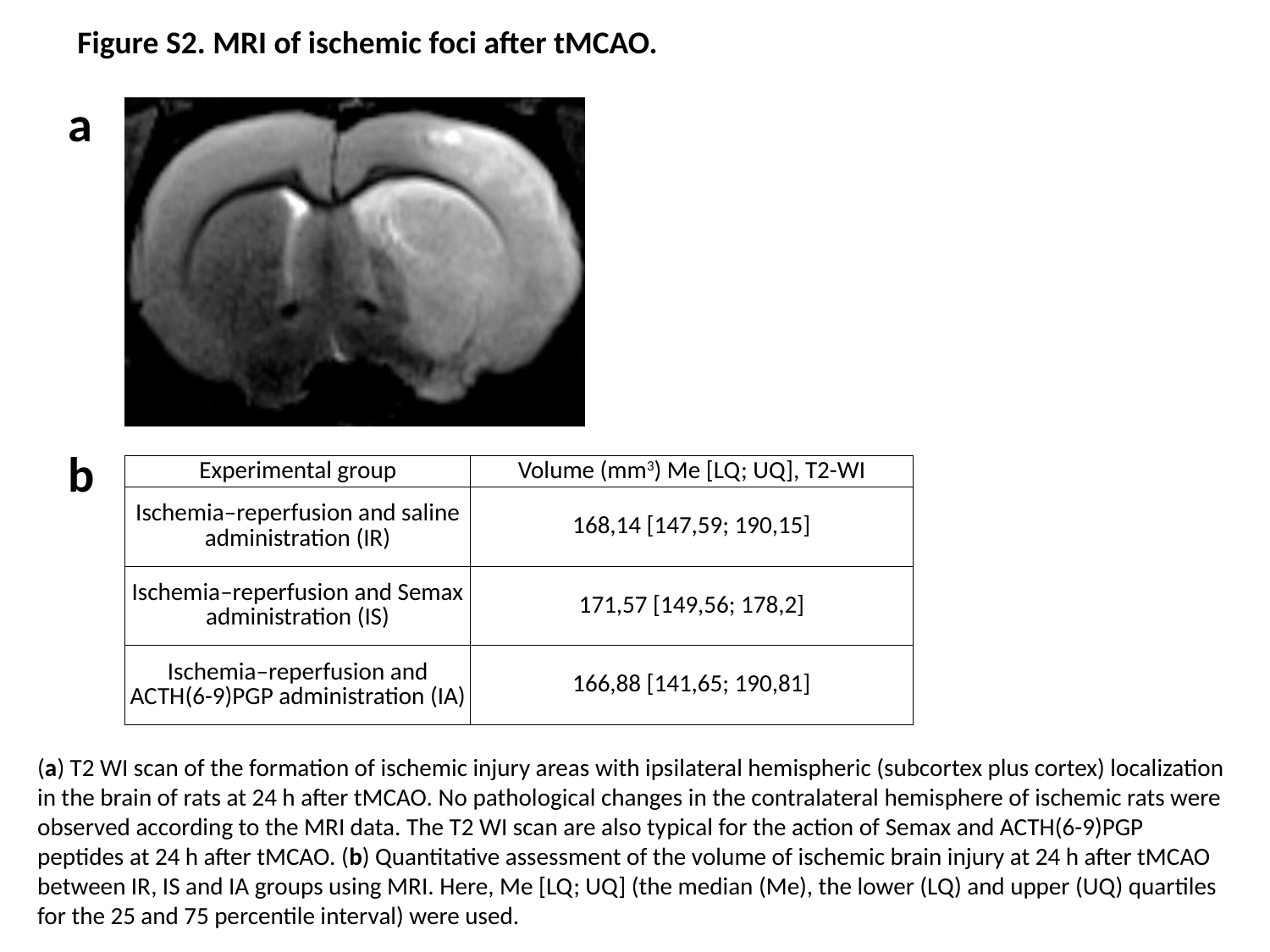

Figure S2. MRI of ischemic foci after tMCAO.
a
b
| Experimental group | Volume (mm3) Me [LQ; UQ], T2-WI |
| --- | --- |
| Ischemia–reperfusion and saline administration (IR) | 168,14 [147,59; 190,15] |
| Ischemia–reperfusion and Semax administration (IS) | 171,57 [149,56; 178,2] |
| Ischemia–reperfusion and ACTH(6-9)PGP administration (IA) | 166,88 [141,65; 190,81] |
(a) T2 WI scan of the formation of ischemic injury areas with ipsilateral hemispheric (subcortex plus cortex) localization in the brain of rats at 24 h after tMCAO. No pathological changes in the contralateral hemisphere of ischemic rats were observed according to the MRI data. The T2 WI scan are also typical for the action of Semax and ACTH(6-9)PGP peptides at 24 h after tMCAO. (b) Quantitative assessment of the volume of ischemic brain injury at 24 h after tMCAO between IR, IS and IA groups using MRI. Here, Me [LQ; UQ] (the median (Me), the lower (LQ) and upper (UQ) quartiles for the 25 and 75 percentile interval) were used.
